# Early Fasting Is Long Lasting: Differences in Early Nutritional Conditions Reappear under Stressful Conditions in Adult Female Zebra Finches

**DOI:** 10.1371/journal.pone.0005015

**Published:** 2009-03-26

**Authors:** E. Tobias Krause, Mariam Honarmand, Jennifer Wetzel, Marc Naguib

**Affiliations:** 1 Department of Animal Behaviour, University Bielefeld, Bielefeld, Germany; 2 Department of Animal Population Biology, Netherlands Institute of Ecology (NIOO-KNAW), Heteren, The Netherlands; University of St. Andrews, United Kingdom

## Abstract

Conditions experienced during early life can have profound effects on individual development and condition in adulthood. Differences in nutritional provisioning in birds during the first month of life can lead to differences in growth, reproductive success and survival. Yet, under natural conditions shorter periods of nutritional stress will be more prevalent. Individuals may respond differently, depending on the period of development during which nutritional stress was experienced. Such differences may surface specifically when poor environmental conditions challenge individuals again as adults. Here, we investigated long term consequences of differences in nutritional conditions experienced during different periods of early development by female zebra finches (*Taeniopygia guttata*) on measures of management and acquisition of body reserves. As nestlings or fledglings, subjects were raised under different nutritional conditions, a low or high quality diet. After subjects reached sexual maturity, we measured their sensitivity to periods of food restriction, their exploration and foraging behaviour as well as adult resting metabolic rate (RMR). During a short period of food restriction, subjects from the poor nutritional conditions had a higher body mass loss than those raised under qualitatively superior nutritional conditions. Moreover, subjects that were raised under poor nutritional conditions were faster to engage in exploratory and foraging behaviour. But RMR did not differ among treatments. These results reveal that early nutritional conditions affect adult exploratory behaviour, a representative personality trait, foraging and adult's physiological condition. As early nutritional conditions are reflected in adult phenotypic plasticity specifically when stressful situations reappear, the results suggest that costs for poor developmental conditions are paid when environmental conditions deteriorate.

## Introduction

Conditions individuals experience during early development have major effects on their developmental trajectory, behaviour, and reproductive capacity [1], [2]. Adverse conditions during early development can have long term effects and major fitness consequences as they can affect phenotypic traits, reproductive success, and survival in several vertebrate species [3]–[8]. Poor nutrition during early development may constrain adult phenotypic plasticity and thus also the flexibility to cope with different environments in adulthood [9]. Understanding causal effects on how individuals acquire and manage resources during such periods of low food availability thus requires assessing to what extent the traits of resource management are affected by nutritional stress during periods of early development. Song birds have been widely used to investigate the consequences of early developmental conditions, as they undergo rapid development from hatching to a fully grown nutritionally independent individual. Yet, most research on effects of early developmental stress on adult traits manipulated rearing conditions for the full first month, covering both, the nestling as well as the fledgling period [10]–[15]. However, a mistiming of breeding or short periods of poor conditions resulting from bad weather is likely to affect only part of the developmental period [16]. Shorter periods of developmental stress, affecting either nestlings or fledglings, thus appear to be of specific ecological relevance. Studies focussing on nutritional stress during the nestling period [17]–[19] revealed long term effects and a recent study addressed the relative importance of the nestling and fledgling period in shaping adult traits [19]. Criscuolo et al [19] showed that adult metabolic rate after food restriction was specifically elevated when birds had experienced nutritional stress as nestling rather than as fledgling and that catch up growth was significantly steeper in subjects that had received nutritional stress as nestlings compared to those that experienced it as fledglings. Differences in nutrition during the different early developmental phases, thus, may affect adult's physiological responses, particularly if poor conditions are re-occurring. This raises the question as to whether such effects are also reflected in behavioural and biometric traits that are linked to the acquisition of resources such as adult body reserve management, foraging, exploration and risk-taking behaviour.

Here we studied the influences of early nutritional conditions on adult responses to short periods of food restriction on body mass regulation and foraging behaviour using adult female zebra finches as model organisms. We manipulated nutritional conditions in fledglings and nestlings in a comparable experimental procedure as Criscuolo et al [19]. Subjects were raised under one of three different experimental treatments. Subjects were raised either on a low quality seed diet during the nestling period (up to day 17) followed by a protein enriched high quality diet during the fledging period (day 17 to 35; LH (low-high) treatment) or they were raised under these nutritional diets in reversed order; hence a high quality until day 17 and a low quality diet from day 17 to 35 (HL (high-low) treatment). A third group was raised under the high quality diet throughout the nestling and fledgling phase (HH (high-high) treatment).

We tested subject's response to food restriction as adults by measuring body mass loss and tested their exploration and foraging behaviour after a short period of food deprivation. We predicted subjects raised on a low quality diet to exhibit lower plasticity and thus are more sensitive to food restriction as adult which consequently leads to faster exploration and to higher body mass loss. As previous studies showed that early development stress leads to an increase in adult metabolic rate [19], [20], we here also included measures of metabolic rate as lowest running nocturnal mean [20]. Criscuolo et al. [19] measured RMR in the morning between 1030 and 1100 hours after subjects were kept without food for more than 17 h, which is longer than in other studies [20]. If differences in RMR among treatment groups are not specific to such more stringent conditions, we predicted that individuals from LH and HL treatments have elevated metabolic rates as adults, compared to individuals from the HH treatment.

## Results

### Experiment 1

**Figure 1:**
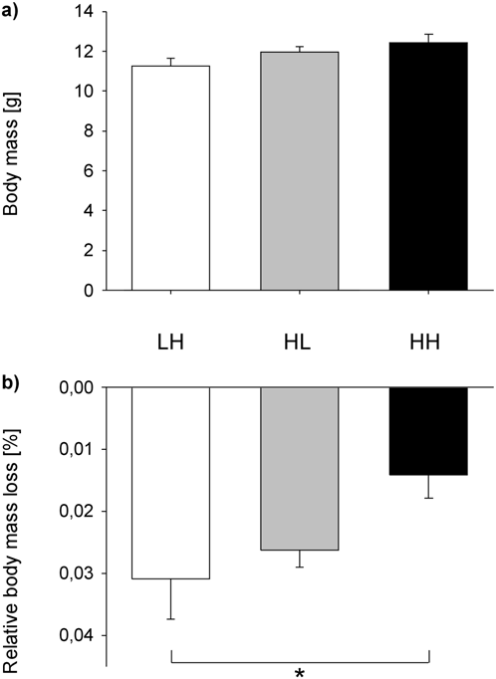
Effects of the nutritional treatments on body mass and relative body mass loss. (a) Mean (±SE) body mass for subjects from the three different nutritional treatments and (b) relative body mass loss during a 3 h food deprivation period for subjects from the different early nutritional treatments, LH, HL and HH. In the LH (low-high) treatment subjects fed on a low quality seed diet until day 17 (nestling period) followed by a protein enriched high quality diet up to day 35 (fledging period). Subjects in the HL (high-low) treatment experienced the nutritional diets vice versa. In the HH treatment (high-high) a high quality protein enriched diet was fed throughout the nestling and fledgling phase (see text for details). * p<0.05.

The three different nutritional treatments during early development, i.e. low quality food during the nestling period and high quality food during the fledging period (LH), high quality food during the nestling period and low quality food during the fledgling stage (HL), and high quality food throughout the nestling and fledgling period (HH), had no significant effect on the body mass of adult subjects (LME: nutritional treatment, F_2,19_ = 2.46, p = 0.11; Fig. 1a).

The relative body mass loss after three hours of food deprivation tended to be different among the three treatments (LME: nutritional treatment, F_2,19_ = 3.35, p = 0.057; age, F_1,7_ = 3.75, p = 0.094; Fig. 1b). Parameter estimates of the final model revealed that LH subjects and HH subjects significantly differed from each other (p = 0.012), and that HL and HH show a tendency to be different (p = 0.0504). LH subjects and HL subjects were not different from each other (p = 0.52). The earlier subjects experienced poorer nutritional conditions during development, the higher their relative body mass loss was (Fig. 1b).

### Experiment 2

**Figure 2:**
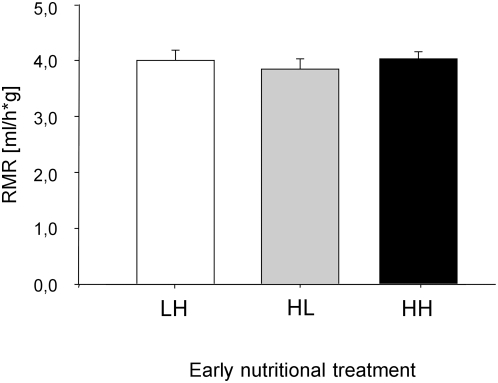
Effects of the nutritional treatments on resting metabolic rate. Mean (±SE) resting metabolic rate (RMR) for subjects from the different early nutritional treatments, LH, HL and HH. In the LH (low-high) treatment subjects fed on a low quality seed diet until day 17 (nestling period) followed by a protein enriched high quality diet up to day 35 (fledging period). Subjects in the HL (high-low) treatment experienced the nutritional diets vice versa. In the HH treatment (high-high) a high quality protein enriched diet was fed throughout the nestling and fledgling phase (see text for details).

Subjects from the three nutritional treatments did not differ in resting metabolic rate (RMR) (LME: nutritional treatment, F_2,22_ = 0.17, p = 0.84; Fig. 2).

### Experiment 3

**Figure 3:**
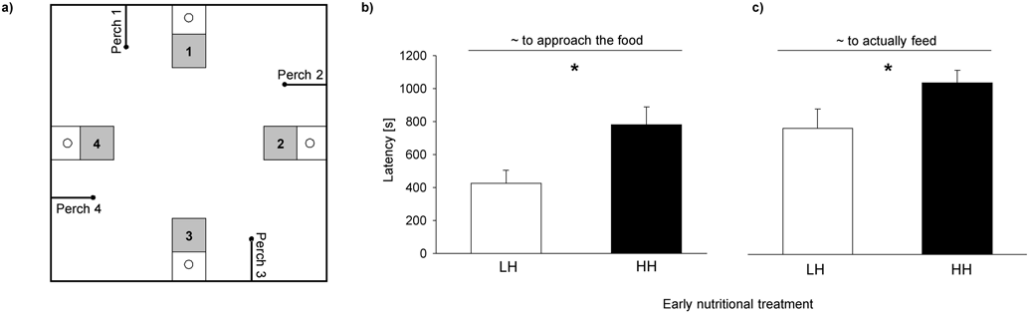
Experimental setup (a) for the spatial foraging test which was conducted in an aviary (1.85×1.85×1.85 m) with four potential feeding sites. The grey areas indicate a hut which blocked the view to the feeding site unless subjects perched on the perch next to the site. The white areas in front of the hut were visible from the adjacent perch and indicate the platform in front of the hut on which the food was provided. Circles indicate the four possible positions of a food dish. During a test only one food dish was rewarded with food, the others remained empty. Drawing is not to scale. (b) The mean latency to approach the food (±SE) for subjects from the LH and the HH treatment and (c) the mean latency (±SE) until subjects fed in this test. In the LH (low-high) treatment subjects fed on a low quality seed diet until day 17 (nestling period) followed by a protein enriched high quality diet up to day 35 (fledging period). In the HH treatment (high-high) a high quality protein enriched diet was fed throughout the nestling and fledgling phase (see text for details). * p<0.05.

Subjects from the LH treatment (N = 13) and from the HH treatment (N = 13) were used to examine effects of early nutritional condition on adult exploration and foraging behaviour. Subjects from the two treatments explored the experimental aviary differently. Subjects from the LH treatment had a lower mean latency to approach the food than subjects from the HH treatment (LME: nutritional treatment, F_1,9_ = 6.05, p = 0.036 Fig. 3b). Body mass was not significant and was excluded from the final model. Subjects from the LH treatment also had a significantly lower mean latency to actually feed compared to subjects from the HH treatment (Mann-Whitney U-test, N_1_ = 8, N_2_ = 8, Z = −2.75, p = 0.006; Fig. 3c).

The analysis on the latency to approach the food in the first trial (when subjects had not yet obtained information about the food location) revealed similar results as the analysis on mean time across all trials (LME: nutritional treatment, F_1,9_ = 6.34, p = 0.033). Body mass was not significant and was excluded from the final model. In the first trial, latencies to actually feed were not significantly different between subjects from the two nutritional treatments (Mann-Whitney U-test, N_1_ = 8, N_2_ = 8, Z = −1.74, p = 0.083).

The mean number of visits throughout all trials at non-rewarded feeding sites prior to feeding was not significantly different between treatments (Mann-Whitney U-test, N_1_ = 8, N_2_ = 8, Z = −0.63, p = 0.53). Also the mean latency to leave the start box did not differ significantly between treatments (Mann-Whitney U-test, N_1_ = 8, N_2_ = 8, Z = −0.84, p = 0.39).

## Discussion

The experiments revealed that female zebra finches experiencing poor nutritional conditions during early development had, as adults, an increased body mass loss after food deprivation, approached food faster and fed earlier than subjects experiencing a high quality diet throughout early development. These body mass losses were the stronger the earlier subjects had experienced poor nutritional conditions during development. The results revealed no effects of early nutritional treatment on resting metabolic rate.

The effects on body mass loss in response to food deprivation may reflect costs that appear to be paid only when adults experience environmental stress. In contrast, such effects were not evident by our measures of resting metabolic rate. These findings seem to contradict predictions made by the thrifty phenotype hypothesis [21]. According to this hypothesis one may have expected subjects from poorer conditions to be better adapted to poor conditions as adults than subjects from better conditions. Possibly, thrifty phenotypes are adaptive only when individuals remain in their natal environments or when natal conditions reliably predict adult environmental conditions, therefore it does not pay to compensate in body mass. This may suggest that thrifty phenotype and catch up growth are two alternative strategies to cope with environmental conditions experienced early in life.

A loss in body mass carries potential costs as individuals losing weight during a short period of fasting have a lower probability of survival [22]. Periods of short food restriction are ecologically relevant as they may occur under natural conditions during bad weather [23], [24]. Such periods may also result from the presence of predators or from social constraints on foraging. Individuals that cope better with periods of food shortage are likely to have a higher probability of survival than individuals that are affected to a greater extent. The higher body mass loss in subjects from poor early conditions may also be interpreted as a reduced level of phenotypic plasticity 9, 25, 26. Thus, constraints during early development may possibly reduce phenotypic plasticity and increase vulnerability to adverse conditions and environmental stress later in life [9], [27].

The lack of an effect of early nutritional conditions on adult metabolic rate contrasts previous studies. Verhulst et al [20] used brood size manipulation to affect conditions experienced during the first 34 days of development in zebra finches which led to a significant increase of metabolic rate in adults from larger broods compared to those from small broods. Our findings that RMR, which was measured in a similar way, was not affected by nutritional stress as nestling or fledgling suggests that birds can compensate for shorter periods of low quality nutrition experienced during development (two weeks in our experiment and five weeks in Verhulst et al. [20]). In contrast to our findings, Criscuolo et al. [19] who imposed nutritional stress during either the nestling or fledgling period, as we did, found an increase in adult metabolic rate in zebra finches raised under a protein poor diet compared to those raised under a protein rich diet. However, in their study RMR was measured as the lowest mean between 1030 and 1100 hours, i.e. after the birds had a long night of about 17 hours without food. Their findings may well have been affected by this longer period subjects had to cope without food and if so, their findings on RMR link well to our findings on exploration and body mass loss where effects of early developmental stress resurfaced after food restriction. There thus seems to be a positive relationship between intensity of actual stress and the nutritional stress experienced during development.

The effects of early nutritional conditions on body mass loss in adults after food deprivation may have been proximately caused by several factors. They may have resulted from an increased metabolism under stressful conditions, as the RMR data from Criscuolo et al [19] suggest. Comparing their results with our study suggests a positive relation between strength of stress and the pronouncement of effects. Another proximate cause could have been differences in crop and intestine fillings. Such fillings could represent an insurance against periods of food restriction even though additional weight can have costs such as reduced flight manoeuvrability and escape behaviour [28], [29]. However, as the mass of faeces represented only about 41% of the total body mass loss, differences in defecation cannot fully explain the differences in mass loss.

Regardless of the proximate causes of body mass loss, such effects are likely to become more pronounced with increasing severity and length of the unfavourable nutritional conditions. Along this line, our findings that subjects explored more rapidly for potential food sites when they had experienced poorer nutritional conditions during early development, also suggest that effects on nutrition in early development reappear in adulthood only when conditions deteriorate. Previous studies showed that zebra finches that had to spend more time foraging had less energy and less time available for reproduction [30], [31] indicating that feeding efficiency is of high fitness relevance [32]. Individuals thus appear to modify their time budget with regard to their energetic state and nutritional needs [33].

Furthermore, the rapid exploration and foraging displayed by subjects that had experienced poorer early nutritional conditions may reflect a more risky foraging strategy to cope with higher nutritional needs. Our data thus suggest that individuals that had experienced poorer nutritional conditions have to deal considerably earlier with allocation problems between nutritional needs and risks of predation than do individuals with a better developmental history [34]. These findings are also in line with previous studies on blue tits (*Cyanistes caeruleus*) [25] and great tits (*Parus major*) [35] showing that exploration behaviour, which is commonly used as a personality trait, can be affected by early development and has fitness effects [36].

Taken together, this study emphasizes how differences in early nutritional conditions in the different developmental phases affect the adult phenotype and its plasticity. The higher sensitivity of nestlings than of fledglings to nutritional stress, suggests that breeding too early, with the risk of missing optimal conditions during early in development, can increase costs for offspring, with respect to the traits measured here. As fledglings appear to be less vulnerable than nestlings, breeding later so that the optimal conditions may fade away does not seem to comprise comparable negative consequence for the offspring. The findings that differences in early nutritional conditions surfaced in adulthood only after food restriction raise questions on how evolution may act on adaptive strategies with which individuals cope with their developmental background. A strategy in which poorer nutritional conditions surface specifically when environmental conditions deteriorate, as shown here, may reflect reduced phenotypic plasticity resulting from poorer developmental conditions.

## Methods

We conducted the experiments from March to June in 2007 at the University of Bielefeld. Subjects were female zebra finches of wild Australian origin. The birds were about the F8 generation in Bielefeld originating from birds obtained from Australia. Molecular analyses also show that these birds cluster with wild birds from Victoria, Australia rather than with the domesticated strains used in most laboratories [37]. Females were obtained from a breeding experiment conducted in 2005 (by M.H.). In this breeding experiment, breeding pairs had received one out of three different nutritional treatments during different stages of their offspring's early post hatching development. Nutritional treatments were either seed mix (here termed: low quality food) or seed mix supplemented daily with protein rich egg (eggfood tropical finches, CéDé, Evergern, Belgium), germinated seeds and, twice a week, greens (here termed: high quality food). In the LH treatment, subjects received the low quality food from day three post hatching until day 17 post hatching and high quality food during the fledging period (until day 35). In the HL treatment, subjects received high quality food from day three until day 17 and low quality food until day 35. In the HH control treatment, subjects received high quality food from day three to day 35. A treatment with seed food from day 3 to day 35 was omitted because breeding success in our zebra finch population is very low when parents are provided exclusively with seed food. From hatching until day three post-hatching, subjects of all treatments received high quality food. From day 35 onwards all subjects received the same diet. This diet was intermediate between the previous food treatments. It consisted of daily ad libitum provisioning with dried and germinated seeds and fresh water (plus vitamins) and egg twice a week.

15 weeks prior to the experiments, all female subjects (N = 37) were captured from free flight aviaries (1.2×3×3 m) and were transferred to cages (83×30×39 cm) with single-sex groups of three to four subjects. In experiment 1 and 2, we used 30 subjects; ten subjects from each treatment (LH, HL, HH). In experiment 3, we used subjects from only two treatment groups (LH and HH). In experiment 3, we used 26 subjects (13 subjects from each of the two treatments), of which 19 subjects were the same as used in experiments 1 and 2.

Subjects had an average age of 546±18 days (mean±SD) at the beginning of the experiments. None of the 37 females had prior breeding experience. The light dark cycle was 16 ∶ 8 hours with light on at 06:00 hours. The mean temperature during experiments was 25.5°C±0.7 (mean±SD) and the humidity was 37%±7. The research was carried out according to the German laws for experimentation with animals and permission to conduct the experiments was granted by the local authorities (Bezirksregierung Detmold).

### Experiment 1 –body mass loss

In this experiment, we measured the relative body mass loss of 30 subjects (10 females from each of the three treatments). All females were subjected to a food deprivation period of three hours, with water available during this period. We measured body mass (Sartorius PT120, measuring accuracy ±0.01 g) of each subject directly before and after this period of food restriction. Relative body mass was calculated as [(body mass before deprivation-body mass after deprivation)/body mass before].

All experiments were started between 0800 and 1000 hours CEST. During the experimental period all subjects were kept in cages of three to four individuals. Subjects from the three treatments were randomly distributed among cages. Subjects were caged in stable social groups throughout the period during which the three experiments were conducted. All subjects in a cage were tested at the same time to reduce additional stress by social isolation. As a consequence we were not able to measure the mass of defecations for each individual separately. Therefore, 10 additional females not otherwise used in the experiments were tested in the same way as described above. However, they were caged individually so that we could measure the mass of defecations for the separate individuals. The body mass of these separated subjects prior to food deprivation was 11.87 g±1.12 (mean±SD). These separated subjects lost an absolute body mass of 0.33 g±0.10. This is a relative loss of 2.8%±0.9 of their initial weight. The mean mass of droppings after the 3 h period of food restriction was 0.12 g±0.10. This equals 40.9%±30.8 of the absolute mass loss.

### Experiment 2 – resting metabolic rate (RMR)

We measured the RMR of the same 30 subjects as in experiment 1 (10 from of each the three treatments (LH, HL and HH)). The RMR was measured as the lowest half-hourly running mean (adapted after [20], [38], [39]). The RMR was measured in consumed O_2_ [ml] per hour and gram body mass. In the metabolic chamber (transparent plexiglas, 18×12×12 cm; Rumed 3501, Rubarth Apparate; Mass Flow Meter FM-360, Tylan Corp.; Oxzilla FC- Oxygene Analyser, Sable Systems) ad libitum food and perches were available. Two subjects were measured simultaneously, each in one of two metabolic chambers connected to the system. The mean temperature of the chambers was 24.3°C (±0.9 SD). Subjects' body mass was measured immediately before placing them in the metabolic chamber. Subjects were placed in the chamber between 1500 and 1700 hours. They were removed between 0900 and 1200 hours. Lights were switched off from 2200 to 0600 hours.

### Experiment 3 – exploration and spatial foraging task

In this experiment, we tested 13 subjects from the LH treatment and 13 subjects from the HH treatment in a spatial foraging task. The tests were conducted in an aviary (1.85×1.85×1.85 m) with four identical feeding sites at a height of 1.35 m (Fig. 3a). Each feeding site consisted of a hut (15×15×15 cm) with one open side to a platform (15×15 cm) in front on which a food dish was placed (Fig. 3a). A perch (length: 30 cm) was located at a distance of 50 cm from each feeding site. Below one feeding site, a start box (20×20×20 cm) was placed 60 cm above the floor. The start box could be opened using a string from outside the aviary. The observer (E.T.K.) was out of sight of the subject and monitored the subject's behaviour on a computer screen connected to two observation cameras (QuickCam Pro 5000, Logitech). Subjects' body mass was measured prior to the experiment.

On the day prior to testing, subjects were familiarised as a group with their cage mates in the experimental aviary for three hours. During this period food was available on the platforms in front of each hut. The following day, subjects where deprived of food for 3 to 4.5 hours before being tested individually in the spatial foraging task. In this task, food (seed mix ∶ egg food, 1∶1) was offered at only one site. The position of this site was altered randomly across subjects. Empty food dishes were placed at the other feeding sites. A test with a given subject consisted of a series of trials in which it was allowed to explore the aviary and to feed from the dish. Before each trial, the subject was given a 5 min acclimation period in the start box. After this period the start box was opened and the subject was allowed to fly into the aviary. Subjects who fed in the aviary were allowed to feed for at maximum 30 sec before being captured with a special caching net. Trials in which subjects did not feed were terminated after 20 min. In both cases, the subject was captured and placed back in the start box, until the next trial started after 5 min. A test with a subject ended when it had fed in four such trials or when it had not fed in four trials in a row. Subjects from the LH treatment did not need more trials (4.9±0.4 trials mean±SE) than did subjects from the HH treatment (4.8±0.4; t-test, t = 0.27, df = 24, p = 0.79). In each trial, we recorded a subject's latencies to (i) leave the start box, (ii) to perch adjacent to the feeding site containing the reward (exploration time) after leaving the start box, and (iii) to actually feed after leaving the start box. Furthermore, we recorded the number of visits at the three empty ‘feeding’ sites.

### Statistical Analysis

Data were tested for normal distribution. Normally distributed data were tested with parametric statistics. When data did not meet normal distribution after transformation we used non parametric statistics.

In experiments 1 and 2, subjects originated from 22 different natal cages and in experiment 3 from 16 natal cages. For non parametric statistics, means of each natal cage were taken into the analysis to avoid pseudo replication. Data of body mass was analysed using a linear mixed effect model (LME) with experimental treatment (variable = categorical, three levels; LH, HL, HH) and age (variable = integer) as fixed factors and natal cage (variable = categorical) as random effect.

Relative body mass loss was analyzed using a LME with treatment and age as fixed factors. For comparisons between the treatment groups parameter estimates are shown as treatment contrasts of the final model. The data for resting metabolic rate were analysed using a LME with nutritional treatment and age at testing as fixed factors and metabolic chamber and natal cage as random factors.

Mean latencies to approach the food in the spatial foraging task were analysed using a LME with nutritional treatment (variable = categorical, two levels, LH and HH) and body mass (variable = continuous) as fixed factors and natal cage as random factor. Latencies to actually feed were analysed using Mann-Whitney U-tests. In addition, as a measure of initial exploration we separately analyzed the latency to approach the food and latency to actually feed in each first trial. The mean number of visits at non-rewarded feeding sites and the mean latency to leave the start box were analysed using Mann-Whitney U-tests. Final models were obtained by using Aikaike's Information Criterion (AIC) to select the most parsimonius model. All models were run in R 2.8.1 software [40], [41] whereas all other statistical analyses were run using SPSS 15.0.1.
